# Supplementary figures and images for: Mitogenome diversity of Aedes (Stegomyia) albopictus: Detection of multiple introduction events in Portugal
Source: PLoS Negl Trop Dis. 2020 Sep 30;14(9):e0008657. doi: 10.1371/journal.pntd.0008657 (PMC7549828; doi:10.1371/journal.pntd.0008657)

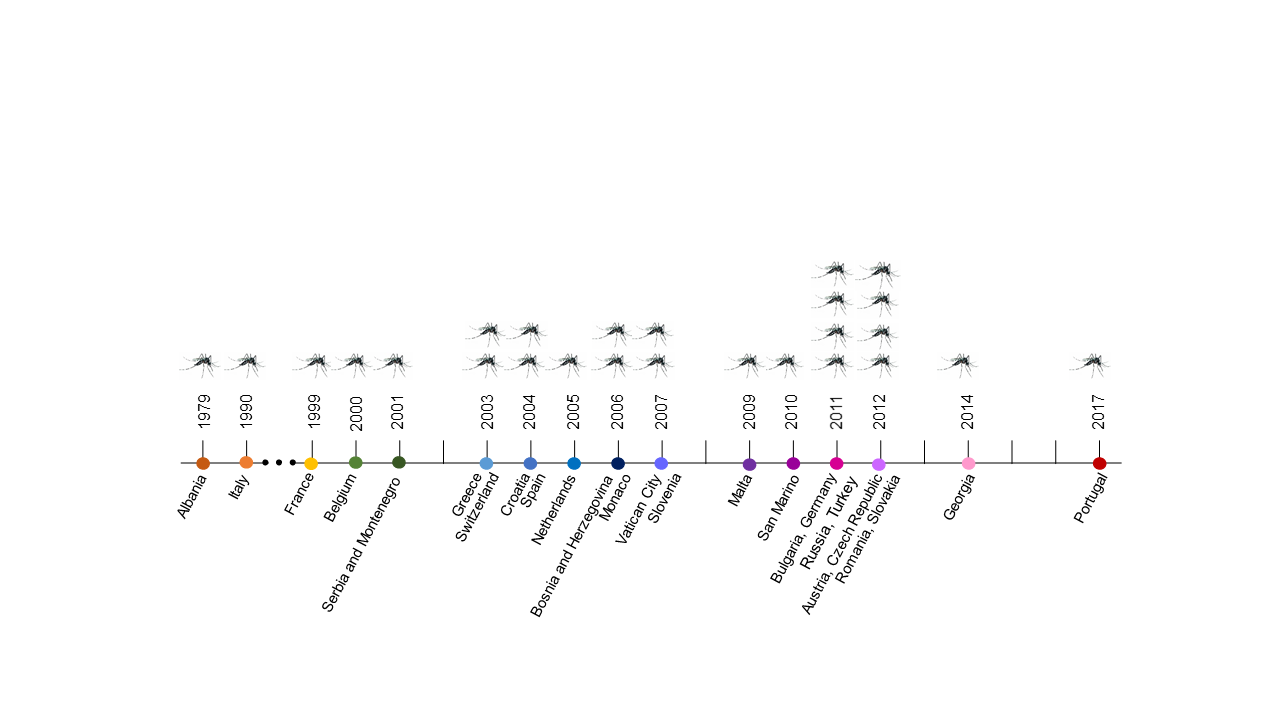

Supplement: S1 Fig — The timeline indicates the years and countries were Ae. albopictus were detected. The number of mosquitos above the line indicates the number of new countries detections at each year. Geographical distribution details in European Centre for Disease Prevention and Control, Aedes albopictus—Factsheet for experts https://ecdc.europa.eu/en/disease-vectors/facts/mosquito-factsheets/aedes-albopictus; Kraemer MU, Sinka ME, Duda KA, Mylne AQ, Shearer FM, Barker CM, et al. The global distribution of the arbovirus vectors Aedes aegypti and Ae. albopictus. Elife. 2015; 4:e08347 https://doi.org/10.7554/eLife.08347; [10–11]. (TIF) [file pntd.0008657.s001.tif]

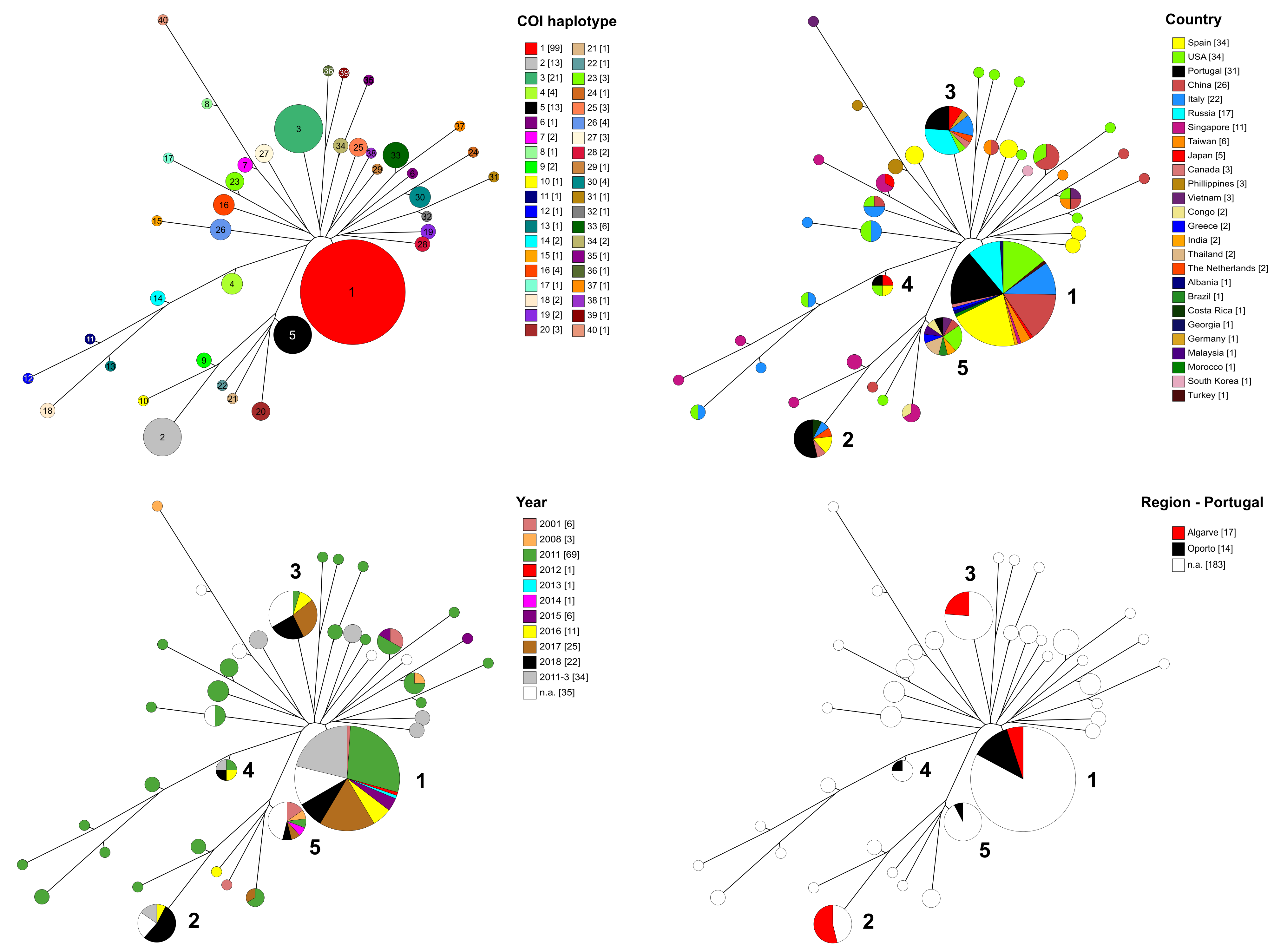

Supplement: S2 Fig — GrapeTree visualization of a maximum likelihood phylogenetic tree constructed based on 31 COI sequences obtained from mosquito circulating in Portugal plus 183 sequences available at GenBank (S1 Table). Tree nodes are colored according with COI haplotype, country, year of collection and geographical region in Portugal. The branch lengths are presented in logarithmic scale and the area of the nodes correlates with the number of sequences with a unique sequence profile (corresponding to a distinct COI haplotypes). The internal region of COI gene under comparison corresponds to positions 1511–2080 of the Rimini isolate 1 reference mitogenome (GenBank accession KX383916). (TIF) [file pntd.0008657.s002.tif]
